# Author Correction: Differential chromatin accessibility in peripheral blood mononuclear cells underlies COVID-19 disease severity prior to seroconversion

**DOI:** 10.1038/s41598-023-33323-8

**Published:** 2023-04-20

**Authors:** Nicholas S. Giroux, Shengli Ding, Micah T. McClain, Thomas W. Burke, Elizabeth Petzold, Hong A. Chung, Grecia O. Rivera, Ergang Wang, Rui Xi, Shree Bose, Tomer Rotstein, Bradly P. Nicholson, Tianyi Chen, Ricardo Henao, Gregory D. Sempowski, Thomas N. Denny, Maria Iglesias De Ussel, Lisa L. Satterwhite, Emily R. Ko, Geoffrey S. Ginsburg, Bryan D. Kraft, Ephraim L. Tsalik, Xiling Shen, Christopher W. Woods

**Affiliations:** 1 Department of Biomedical Engineering, Pratt School of Engineering, Duke University, Durham, NC 27708 USA; 2 Center for Applied Genomics and Precision Medicine, Duke University School of Medicine, Durham, NC 27710 USA; 3 Durham Veterans Affairs Health Care System, Durham, NC 27705 USA; 4 Division of Infectious Diseases, School of Medicine, Duke University Medical Center, 40 Duke Medicine Circle, Durham, NC 27710-4000 USA; 5 Department of Pharmacology and Cancer Biology, School of Medicine, Duke University, Durham, NC 27710 USA; 6 Institute for Medical Research, Durham, NC 27705 USA; 7 Department of Molecular Genetics and Microbiology, School of Medicine, Duke University, Durham, NC 27710 USA; 8 Duke Human Vaccine Institute and Department of Medicine, School of Medicine, Duke University, Durham, NC 27710 USA; 9 Department of Civil and Environmental Engineering, Pratt School of Engineering, Duke University, Durham, NC 27708 USA

Correction to: *Scientific Reports* 10.1038/s41598-022-15668-8, published online 09 July 2022

The original version of this Article contained errors, where a study participant was omitted in the mild COVID-19 experimental group for “bulk” analysis. As the result, in the Results, under ‘A clinical cohort to study early COVID-19 with mild or moderate symptoms’,

“Subjects with mild symptoms exhibited a mean score of 12.9 ± 2.1 and 12.2 ± 2.8 for total PBMC assays (bulk) and assays of individual PBMCs (single-cell assays), respectively, which corresponded to the World Health Organization (WHO) Ordinal 8-point scale 1 (OS1, ambulatory and with no limitation of activities).”

now reads:

“Subjects with mild symptoms exhibited a mean score of 12.8 ± 1.9 and 12.2 ± 2.8 for total PBMC assays (bulk) and assays of individual PBMCs (single-cell assays), respectively, which corresponded to the World Health Organization (WHO) Ordinal 8-point scale 1 (OS1, ambulatory and with no limitation of activities).”

In Table 1, in column ‘Mild disease’, the 'Number of subjects', 'Sex (male/female)' and ‘Max severity score (± SE)’ have been corrected. The correct and incorrect values are listed below.

Incorrect:

**Table:** 

Characteristics	Mild disease
Number of subjects	7
Sex (male/female)	4/3
Max severity score (± SE)	12.9 ± 2.1

Correct:

**Table:** 

Characteristics	Mild disease
Number of subjects	8
Sex (male/female)	5/3
Max severity score (± SE)	12.8 ± 1.9

Additionally, the same error occurred in Supplementary Information Figure S1 and Supplementary Table S1.

The original Supplementary Figure S1 and accompanying legend appear below.

**Figure 1:**
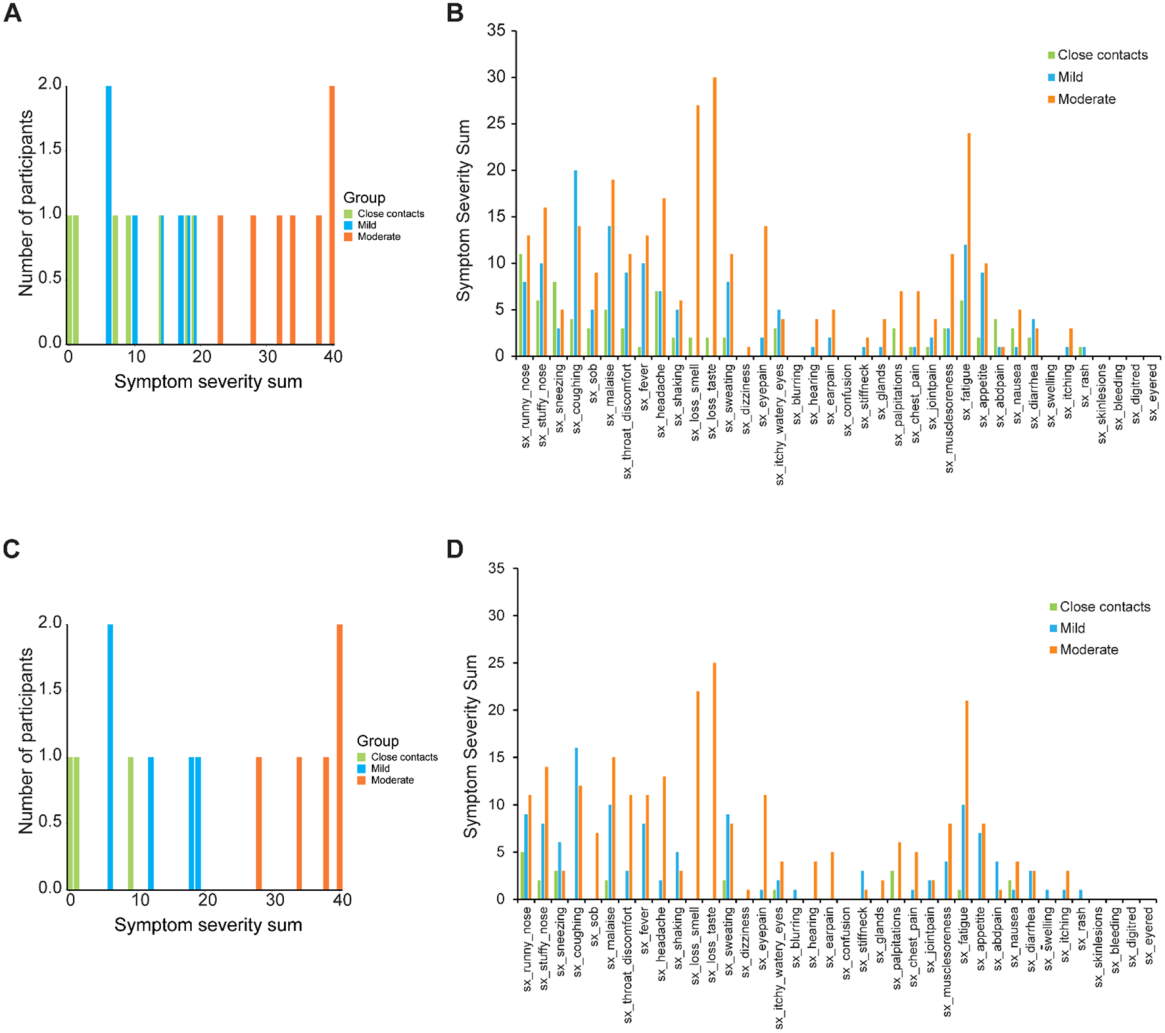
(**A**) Symptom severity sums for the subjects profiled with bulk assays and (**B**) severity scores for each reported symptom for the subjects profiled with bulk assays. (**C**) Symptom severity sums for subjects profiled with single-cell assays and (**D**) severity scores for each reported symptom for the subjects profiled with single-cell assays. For both cohorts, the sum of severity scores for all reported symptoms are shown for close contacts (green), COVID-19 subjects with mild symptoms (blue), and with moderate symptoms (orange).

A new row has been added to Table S1. It now reads:

**Table:** 

Group	Alias ID	Race	Ethnicity	Day of PBMC collection			PCR			Serology Test (IgG)		
Mild	7085CA	White	Non-Hispanic	0	7	14	N^‡^	N	N	N	N	P

The original Article and Supplementary Information files have been corrected.

